# Dynamics of immunity over time: decline of anti-SARS-CoV-2 IgG antibodies and T-cell responses after mRNA vaccination in residents and health care workers in nursing homes and homes with assisted living support

**DOI:** 10.3205/id000082

**Published:** 2023-09-06

**Authors:** Julia Schiffner, Nora Eisemann, Hannah Baltus, Sina Jensen, Katharina Wunderlich, Stefan Schuesseler, Charlotte Eicker, Bianca Teegen, Doreen Boniakowsky, Werner Solbach, Alexander Mischnik

**Affiliations:** 1 Health Protection Authority, Luebeck, Germany; 2 Institute of Social Medicine and Epidemiology, University of Luebeck, Germany; 3 Klinisch-Immunologisches Labor Stoecker, Luebeck, Germany; 4 Vorwerker Diakonie gemeinnuetzige GmbH, Luebeck, Germany; 5 Center for Infection and Inflammation Research, University of Luebeck, Germany; 6 German Center for Infection Research (DZIF), Luebeck, Germany

**Keywords:** COVID-19, SARS-CoV-2, immunoglobulin, IgG, IgA, T cell immunity

## Abstract

**Background:**

In the present study, we investigated the dynamics of immunity over time by measuring anti SARS-CoV-2 IgG antibodies and SARS-CoV-2 specific T-cell responses (interferon-gamma release assay) after two doses of vaccines in residents and health care workers (HCW). Mostly, 224 (98%) residents and 244 (89%) HCW received two doses of mRNA vaccine (BNT162b2, Pfizer-BioNTech); the rest of the participants received heterologous vaccinations with mRNA and vector vaccines. The study was conducted at the time when the Delta variant of SARS-CoV-2 prevailed.

**Methods:**

We analyzed blood samples of 228 residents (median age 83.8 years) and of 273 HCW (median age 49.7 years) from five nursing homes and one home for the elderly with assisted living support at one specific time point. Participants received two vaccinations. The blood samples were analyzed for SARS-CoV-2 specific IgG antibody and T-cell responses.

**Results:**

The initial immune responses in the younger participants were about 30% higher than in the older age group. Over time the estimated mean of the parameters (estimated from the study sample for the total population) decreased in all groups within the maximum observation period of 232 days. Comorbidities such as coronary heart disease or diabetes mellitus reduced the initial immune responses regardless of age. With regard to measured IgG antibody levels, absolute values decreased over time, whereas the interferon-gamma response remained at a constant level between day 120 and 180 and seemed to be less dependent on the time elapsed after vaccination.

**Conclusions:**

Based on our data, it does not seem possible to determine a reliable threshold of robust immunity, but we suggest that high titres of neutralizing capacity and interferon-gamma response might be an indicator of protection against severe COVID-19 courses.

## Introduction

Multiple COVID-19 vaccines have been developed that offer protection against severe course of disease by generating immune responses against the spike antigen of SARS-CoV-2. In Germany, the national vaccination program started with the Pfizer-BioNTech BNT162b2 mRNA vaccine (B/P Comirnaty) on December 27, 2020, followed by the approval of Spikevax mRNA (mRNA; Moderna) on January 6, 2021, and ChAdOx1 nCoV-19 vector vaccine (Vaxzevria; AstraZeneca (AZ)) on January 29, 2021 [1]. Initially, vaccines were administered to priority groups, including residents of old people’s and nursing homes, persons aged ≥80 years, personnel with a particularly high risk of exposure in medical facilities (e.g. in emergency rooms or in the medical care of COVID-19 patients), personnel in medical facilities with close contact to vulnerable groups (e.g. in oncology or transplant medicine), nursing staff in outpatient and inpatient care for the elderly, other workers in homes for the elderly and nursing homes with contact to residents. Later, vaccines were recommended population-wide for each individual ≥6 years. 

It has been reported by our group [2], [3] and others [4], [5], [6] that the cellular and humoral immune response wanes over time after infection and also after vaccination. In the present study, we were primarily interested how anti SARS-CoV-2 IgG antibodies and SARS-CoV-2 specific T-cell responses declined over time after two doses of mRNA vaccines, especially by age-group and comorbidity status. We analyzed the long-term course of the immune response with respect to serum IgG antibodies and the capacity of peripheral blood cells to produce interferon-gamma upon viral S-protein specific stimulation. We did not compare individual antibody titers over time. Rather, we collected one sample per patient and considered the time elapsed since the last vaccination.

Recently, several papers comment on waning humoral immune response after SARS-CoV-2 vaccination [7]. A recent systematic review on a threshold of humoral immunity suggests that high titres might correlate with robustness of protection [8]. In comparison to many other studies our present work focused on determining humoral and T-cell based immunity at the same time after twofold vaccination.

The recommendations for vaccination at the time of the study did not rely on individual reactivity of the immune system but solely on protection data in large cohorts. What is missing is a clinical parameter to support practitioners in deciding if an individual is protected or not. The need of a booster vaccination is extensively documented in various studies [9], but the currently recommended timepoint – from three months after the second vaccination – might not be appropriate for the individual person.

## Material and methods

### Study population

The study includes persons either living or working in six old people’s homes in Northern Germany. Because of the study’s exploratory nature, we aimed for the largest possible sample size that was affordable. The recruitment took place in five facilities that are stationary retirement homes and one facilitiy that is a so-called assisted living home. The study period was between August 31 and September 9, 2021.

**Figure 1:**
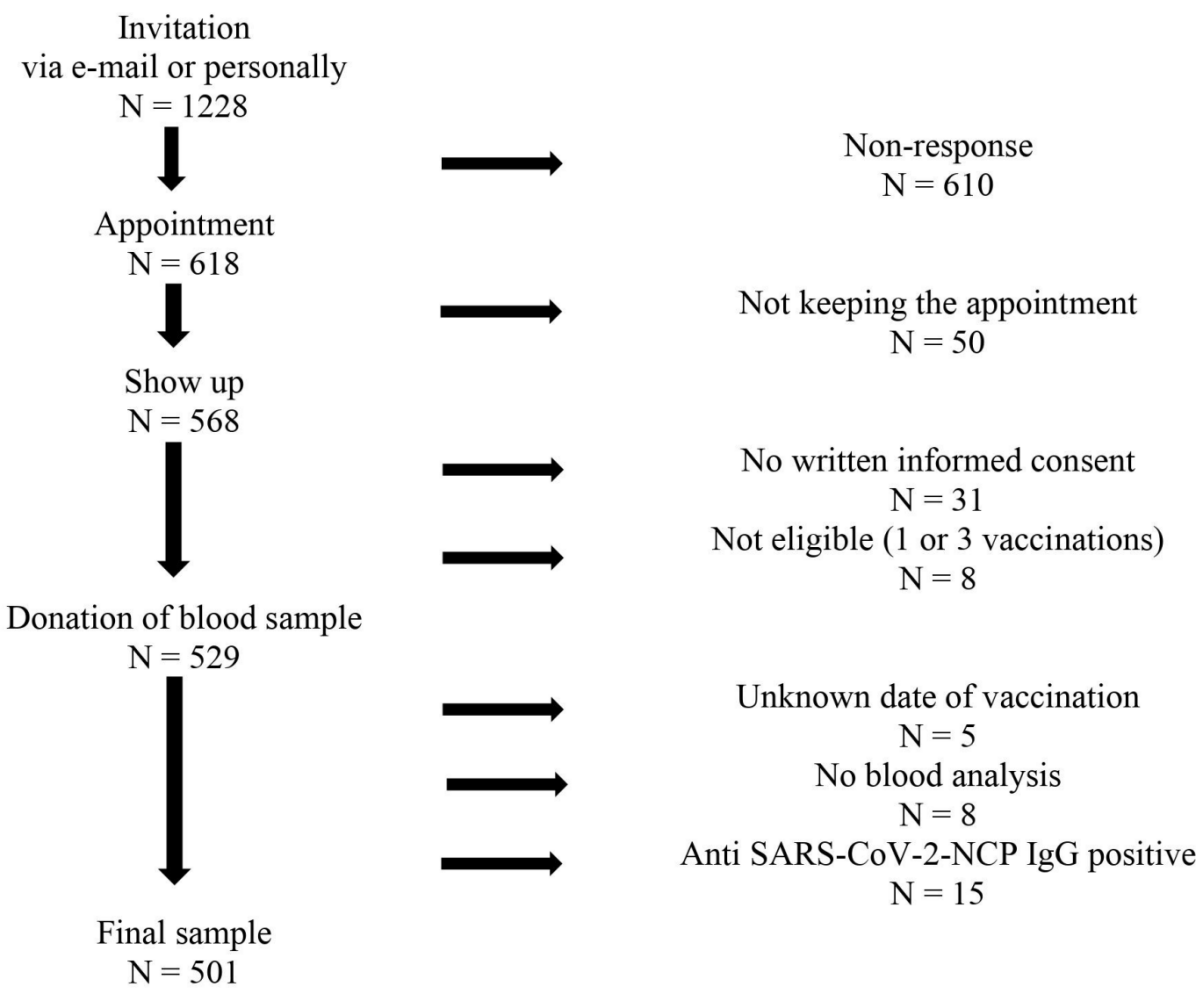
Flow chart of the ISCOV-VAC study recruitment

In total, 1,228 persons were invited by e-mail or personal contact to participate. Inclusion criteria were being vaccinated twice against SARS-CoV-2, an elapsed period of at least 14 days since the second vaccination (as the vaccination effect is first built up during this time) and written informed consent. Exclusion criteria were a third vaccination, unknown date of second vaccination, unsuccessful blood drawings, no laboratory result of the blood sample or a positive test for anti-SARS-CoV-2 nucleocapsid protein (NCP) antibodies. Such a positive test may indicate an undetected infection, which would bias the results. Figure 1 (Fig. 1) shows the flow-chart of recruitment. 

At the study visit, blood samples were taken from the participants and transferred directly to the laboratory within four hours. In addition, the participants filled in a questionnaire on personal data (e.g. age, sex, body height and body weight) and comorbidities (such as diabetes, autoimmune diseases, cardio-vasculary disease). 

### Laboratory methods

The blood samples were analyzed for four main outcomes: anti-SARS-CoV-2 S1-protein IgG antibodies, antibody neutralization capacity, SARS-CoV-2 S1 reactive T cells (i.e. interferon-gamma release assay, IGRA) and anti-SARS-CoV-2 nucleocapsid protein antibodies. 

#### Detection of anti-SARS-CoV-2 S1-protein IgG antibodies

Serum IgG antibodies against the viral (strain Wuhan-1) S1 domain of the spike protein including the receptor binding domain (RBD) were detected by using the “Anti-SARS-CoV-2 QuantiVac ELISA” detection kit (EUROIMMUN; Luebeck, Germany, product no. EI 2606-9601-10 G) according to the instructions of the manufacturer. The measured “relative units/ml” were calibrated with the “First WHO standard of anti-SARS-CoV-2 immunoglobulin” (NIBSC code 20/136) and converted into Binding Antibody Units (BAU)/ml by multiplication with the factor 3.2. Interpretation is as follows: <25.6 BAU/ml=negative; ≥25.6 BAU/ml=positive.

#### Detection of antibody neutralization capacity

Antibody binding of SARS-CoV-2-S1/RBD neutralizing antibodies was detected by applying the “Anti-SARS-CoV-2 NeutraLISA” detection kit (EUROIMMUN; Luebeck, Germany, product no. EI 2606-9601-4) according to the instructions of the manufacturer. This is a surrogate neutralization test which has 98.6% concordance when compared to plaque-reduction (PRNT_50_) testing. Specificity is 99.7% and sensitivity is 95.9%. Values are interpreted as follows: <20%=negative; 20%–<35%=borderline; ≥35%=positive. 

#### Determination of SARS-CoV-2 S1 reactive T cells (interferon-gamma release assay, IGRA)

T cells in peripheral blood reacting to SARS-CoV-2 S1 protein were detected by using the “Quant-T-Cell ELISA” (EUROIMMUN; Luebeck, Germany product no. EQ 6841-9601 and ET 2606-3003). In brief, heparinized blood cells were cultured with S1 antigen for 24 hours. Subsequently, interferon-gamma release was determined in the culture supernatant by ELISA.Values are expressed in mIU/ml. Interpretation is as follows: <100 mIU/ml=negative, 100–200 mIU/ml=borderline; ≥200 mIU/ml=positive.

#### Detection of anti-SARS-CoV-2 nucleocapsid protein (NCP) antibodies

To discriminate between vaccine-induced antibody response and convalescent SARS-CoV-2 infection, serum IgG antibodies against the nucleocapsid protein were detected by using the “Anti-SARS-CoV-2 NCP ELISA” detection kit (EUROIMMUN; Luebeck, Germany product no. EI 2606-9601-2 G) in a semi-quantitative manner. Values are given in ratios. Ratios are calculated from the extinction of the sample and that of a standardized calibrator. Interpretation of values was done as follows: <0.8=negative; ≥0.8–<1.1=borderline; ≥1.1=positive. 

The tests yielded 15 positive results. These individuals were excluded from the study. The validity and reliability test characteristics have been described recently [10].

### Statistical methods

Anti-SARS-CoV-2 S1-protein IgG antibody values above 384 BAU/ml were reported as ‘above 384’ by the laboratory and conservatively set to 385 for further analysis, as was the case with values reported as ‘below 3.2’, which were set to 1.6. Similarly, SARS-CoV-2 S1 reactive T-cell values above 2,500 mIU/ml were set to 2,500 mIU/ml. Pairwise correlation coefficients were calculated for SARS-CoV-2 S1 reactive T cells, anti-SARS-CoV-2 S1-protein IgG antibodies and antibody neutralization capacity. As the Spearman correlation between anti-SARS-CoV-2 S1-protein IgG antibodies and antibody neutralization capacity was very high, the anti-SARS-CoV-2 S1-protein IgG antibody data appeared dispensable and we continued only with SARS-CoV-2 S1 reactive T cells and antibody neutralization capacity. The time after second vaccination was measured on a continuous scale. The values of interferon-gamma and of neutralizing capacity were each plotted against the number of days since second vaccination. Local polynomial regression models (with linear polynomials) were fitted to describe the change in the outcomes over time, together with the corresponding 95% prediction intervals. Subgroup analyses by age (age below 65 years versus 65+), sex (female versus male) and comorbidity (no versus any comorbidity) were performed. Local polynomial regression models (with linear polynomials) were used. All analyses were conducted with R 4.1.1 [11].

## Results

### Characteristics of the study participants

We analyzed blood samples of 228 residents (median age 83.8 years) and 273 health care workers (HCW; median age 49.7 years) from five nursing homes and one home for the elderly with assisted living support. 

Of the final 501 study participants, 273 were HCW (in the retirement homes) and 228 were seniors living in the facilities. A description of the characteristics of the study population is given in Table 1 (Tab. 1). The age of the participants ranged between 19 and 100 years, with a median age of 83.8 years in residents and 49.7 years in HCW. The majority of residents and HCW was female (67% and 75%, respectively). The body mass index (BMI) was in the normal range for 50% of the residents and 39% of the HCW, while few participants were underweight (3.9% and 1.5% in residents and HCW, respectively) and most were overweight or obese (47% and 59% in residents and HCW, respectively). Comorbidities were very common among residents (96%) but also among HCW (54%). The most frequent comorbidities were coronary heart disease in residents (79%) and HCW (25%) and neurological disease in residents (48%). Diseases of the immune system (not specified) were mentioned in about 20% of both residents and HCW. Only 8 patients regularly received immunosuppressive treatment including steroids or TNF alpha blockers.

Different combinations of vector (AstraZeneca) and mRNA (B/P and Moderna) vaccines were possible, but the vast majority received B/P as first and second vaccine (98% in residents and 89% in HCW). The first vaccination was given between December 24, 2020, and June 29, 2021, and the second between January 19, 2021, and August 10, 2021. The time between second vaccination and blood sampling ranged from 22 to 232 days. No data was collected for the first three weeks after second vaccination because the natural immune response takes about two weeks. We did not observe any symptomatic breakthrough infection during the observation period. 

### Immune responses

**Figure 2:**
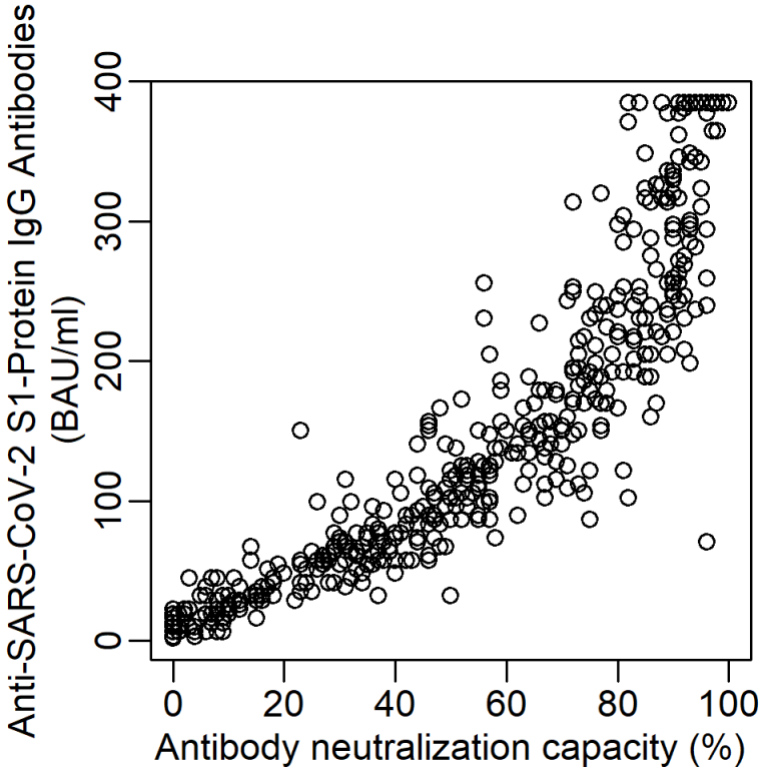
Bivariate scatterplot for Anti-SARS-CoV-2 S1-Protein IgG antibodies and neutralisation capacity (N=501)

**Figure 3:**
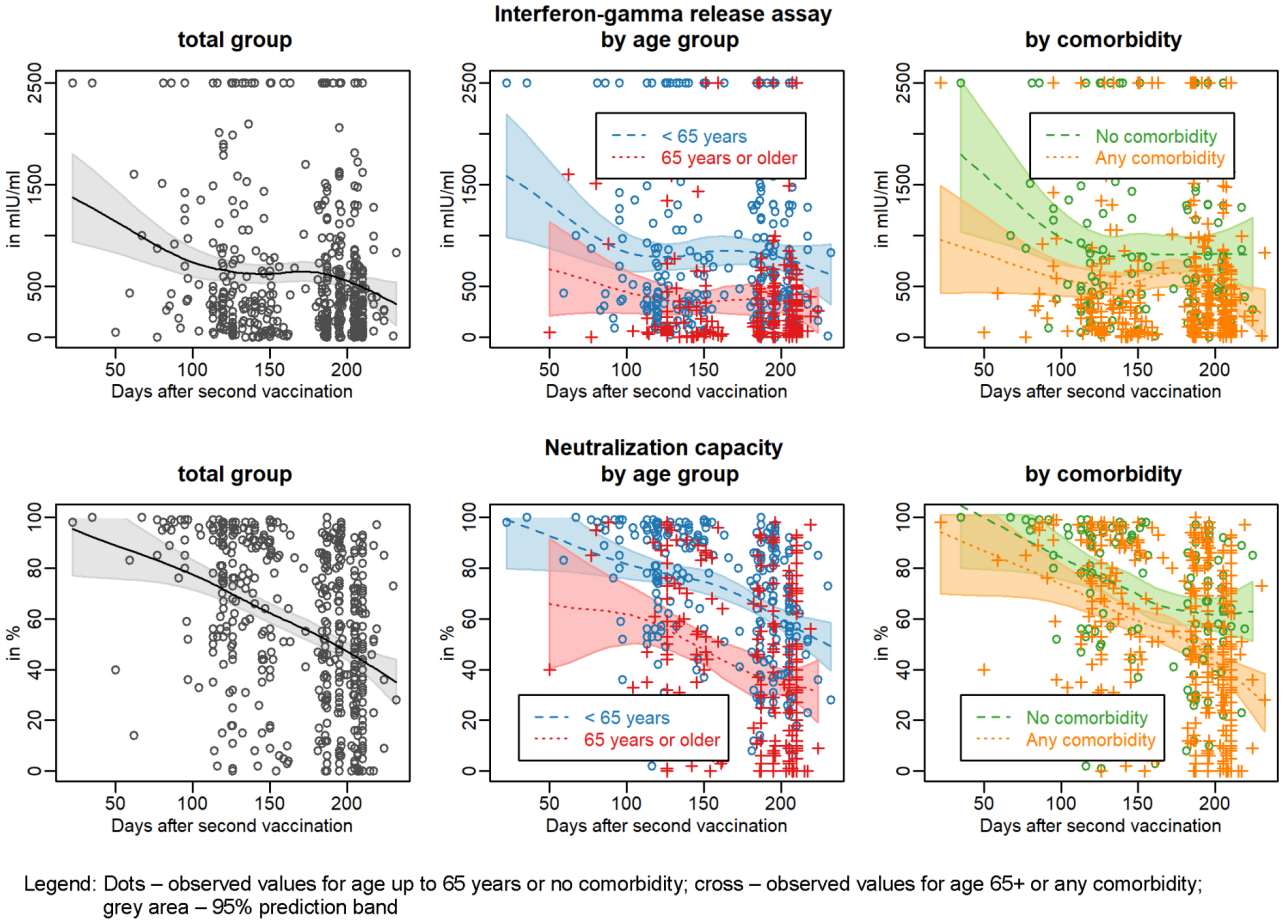
SARS-CoV-2 S1 reactive T cells and neutralisation capacity over time in the total group by age group and by comorbidity status

First, we determined how the anti SARS-CoV-2 IgG antibody levels corresponded to the neutralising capacity. The bivariate scatterplot in Figure 2 (Fig. 2) shows a high correlation (Spearman correlation of 0.959, 95% confidence interval [0.951 to 0.966]). Thus, for further considerations we concentrated on the neutralizing capacity. Pairwise scatterplots and correlation coefficients between all three outcomes are given in Figure S1 in Attachment 1. If we look at the dynamics of the neutralising antibodies, Figure 3 (Fig. 3) (lower panel) shows an almost linear decrease over the entire study period. After about 200 days after two doses of the vaccine, the neutralisation capacity had dropped from >90% to about 40%.

For the subgroup analysis, we formed two age groups: persons under 65 years of age, which included mostly the HCW, and those over 65 years of age, i.e. the residents of the old people’s homes. As expected, shortly after the second vaccination the antibody response was only almost half as high in the elderly group as in the younger group (Figure 3 (Fig. 3), lower panel, middle). However, the relative decline over time was comparable. It is therefore not surprising that the duration of the protective effect of vaccination is limited in the elderly. We were then interested in seeing how the comorbidities reported by the subjects were reflected in the antibody response. The predicted neutralisation capacity is slightly lower for persons with existing comorbidities at any time. The kinetics of the antibody decrease were comparable (Figure 3 (Fig. 3), lower panel, right). The low number of study participants did not allow any meaningful conclusions to be drawn about the effect of any single comorbidity (data not shown). Further subgroup analyses did not show relevant differences regarding sex or BMI category. Subgroups were too small (and in some cases observations were too differently distributed over time) to make reliable comparisons by care level, vaccine or individual diseases. 

When looking at the T-cell response with regard to spike-protein specific interferon-gamma secretion IGRA, it is noticeable that the values also drop linearly between about 50 and about 120 days after the second vaccination but then remain on a plateau (approx. 700 mIU/ml) until about 180 days, after which they drop further (Figure 3 (Fig. 3), upper panel, left). Here, too, there is a clear difference between the older (65+ years) and younger participants (<65 years). In the elderly, the T-cell reactivity was only about 50% in comparison to the younger participants (Figure 3 (Fig. 3), upper panel, middle). The average value of interferon-gamma halved 111 days after second vaccination (95% prediction interval: 80 to 190 days) and the antibody neutralization capacity did so after 199 days (95% prediction interval: 189 to more than 232 days).

A division of the study participants into those with and without comorbidities showed a similar picture as in the analysis of the antibodies. Comorbidities of any kind led to reduced T-cell reactivity. It is therefore plausible to assume that the duration of the protective effect against SARS-CoV-2 infection is also limited. Our data show and confirm the data of others that the immune response after two vaccinations varies greatly from individual to individual but clearly diminishes within the observation period of up to 232 days. 

## Discussion

Many observational studies in which the course of the vaccine efficacy of the COVID-19 vaccines is analyzed show that over a period of 4–6 months after completion of the basic immunisation there is only a slight decline in efficacy against severe COVID-19 disease (hospitalisation). The decline in efficacy against symptomatic infections of any severity, on the other hand, is more pronounced in most studies and amounts to between 10% and 50% (depending on the vaccine and age group) [12]. In consequence, a third vaccination, usually 3 months after the second vaccination, is recommended in most countries to booster the immune system [13].

In our study, we compared the humoral and cellular immune response after two vaccinations of residents of nursing homes over 65 years of age with that of equally vaccinated under-65-year-old employees in these facilities. At the time of the study, the Delta variant prevailed (98%). Double vaccination with mRNA vaccines provides less protection against infection with the Delta variant. The protection against a severe course is still very high. As of December 14 and since December 13, 2021, only 101 SARS-CoV-2 Omicron variant of concern (VOC) cases have been confirmed in Germany [12]. We were able to show that, on average, the initial immune responses in the younger participanta were about 30% higher than in the older ones. Over time, all parameters dropped continuously in all groups within the maximum observation period of 232 days. The existence of any comorbidities such as coronary heart disease or diabetes mellitus reduced the initial immune responses, regardless of age. These data support and extend the findings by Delbrück et al. [9] and clearly demonstrate the need for a third vaccination. Interestingly, in contrast to a tendency to lower values of IgG antibody levels, we observed that the interferon-gamma response remained at a constant level between about day 120 and 180, only to decline further thereafter (Figure 3 (Fig. 3)). This might reflect a twofold reaction from the T-cell compartment. In the first wave after vaccination, primary T cells with limited longevity are stimulated to produce interferon-gamma, followed by a second wave of long-lived T memory cells that compensate (between 120 and 180 days) for the further drop in interferon-gamma levels [14].

Our data thus show that measurable immune parameters may decline within months, accordingly resulting in increasing risks for breakthrough infections in health care workers and in the general population [15]. Little is known about what the measurable immunological values mean for the protection of the individual. Although most of the currently accepted correlates of protection are based on antibody measurements, there is currently no validated threshold value for protection from SARS-CoV-2 infection, albeit it is urgently needed [16], [17]. However, an association between anti-S1 RBD IgG and neutralization antibody levels after immunization with BNT162b2 has been reported [18], [19], [20], [21]. At the time of the study, the messaging from regulatory agencies states that antibody tests should not be used to evaluate a person’s level of immunity or protection from COVID-19 [22]. The attempt to set a clear threshold value for protection against a severe course of COVID on the one hand and a limit value for the general prevention of infection on the other has been unsuccessful in recent years.

Although seroprevalence is currently used as a crude measure of community immunity, having a correlate of protection would allow more precise estimations that could then trigger interventions such as vaccination campaigns if the percentage of immune individuals is deemed to be too low.

Finally, there are many participants, who are recovered from COVID-19 and have high antibody levels for a long time [3]. Chau et al. [23] report a median of 91.1% and an interquartile range of 77.3% to 94.2% for neutralizing antibody levels in vaccinated individuals who remained uninfected. Feng et al. [18] determined that an IgG antibody level of at least 264 BAU/ml is associated with an 80% vaccine efficacy against primary symptomatic COVID-19. For T-cell parameters, so far no studies make a link of titres to the level of protection. A one-time measurement can necessarily only be a snapshot. To estimate the continuity of the protection status, the examinations must be repeated regularly, approximately at intervals of two to three months. In practice, analysis of the T-cell response may not be necessary (Figure S2 and Figure S3 in Attachment 1). Conversely, we were able to show that a higher threshold affects the estimated proportion of people protected (Figure S4 in Attachment 1).

### Lacking evidence of correlate of protection

**Figure 4:**
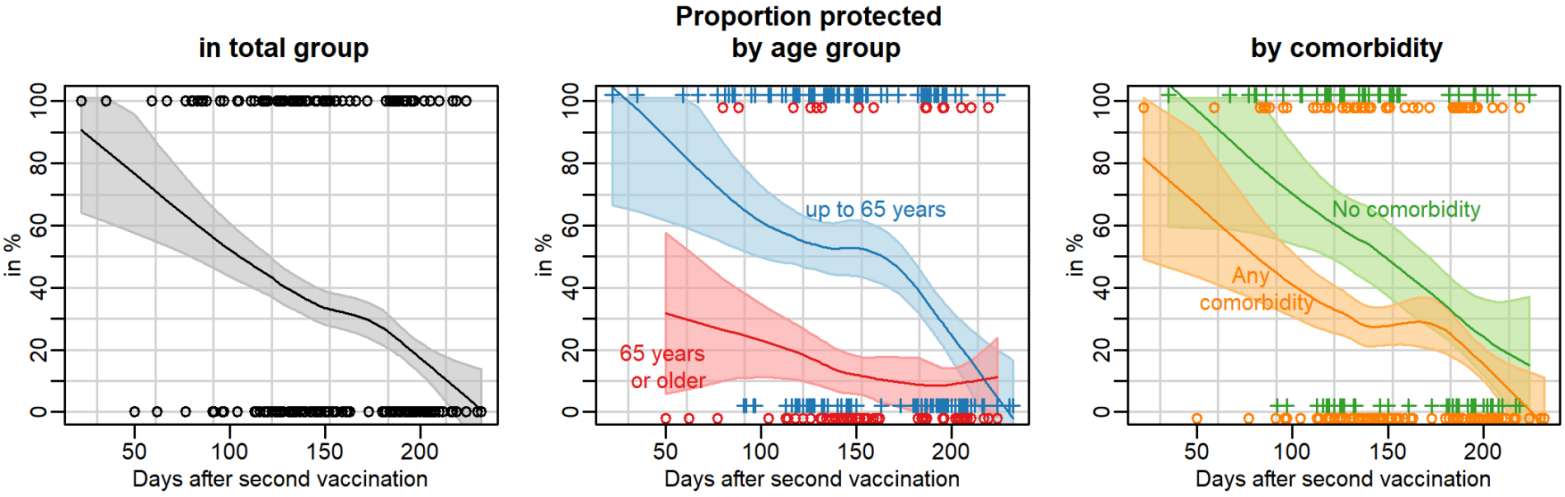
Proportion of persons that are protected against COVID-19 disease over time after second vaccination in the total group by age group and by comorbidity status. A person is considered to be protected against COVID-19 if the SARS-CoV-2 S1 reactive T-cell test is positive, i.e. >200 mIU/ml, and the neutralisation capacity is >75%. Dots and crosses indicate the individual protection status (0%=not protected, 100%=protected), the line indicates the predicted proportion and the grey area indicates the 95% prediction band.

For many technical and medical reasons, there is currently no immunological parameter that allows a reliable statement about the protection status against COVID-19 disease for the individual. Although numerous studies suggest a strong correlation between neutralizing antibody levels and protection [12], many of the regulatory agencies state that antibody tests should not be used to evaluate a person’s level of immunity or protection from COVID-19. This is highly unsatisfactory but may be difficult to determine. At the time of the study, a person living in Germany who had recovered from COVID-19 more than three months ago had many restrictions in everyday life, irresprective of documented high antibody levels and high IGRA levels. We have therefore analyzed the data collected here to estimate what the course of protection would be depending on the time passed after the second vaccination. Based on these assumptions, it appears that the vast majority of persons (95%) can be assumed to be protected three weeks after the second vaccination, although the small sample size at this observation time causes a quite large amount of uncertainty, as indicated by the wide 95% prediction interval (Figure 4 (Fig. 4)). The proportion of protected individuals decreases continuously over time. Fifty percent of persons are still protected 106 days after second vaccination. Younger individuals under 65 years are protected for a longer time; on average, it takes 164 days until only 50% are still protected, while the proportion of protected older individuals is always below 50%. Those without comorbidity are protected up to 50% for 149 days after second vaccination and those with comorbidity for 81 days. 

We are convinced that increasing neutralization capacity might correlate with robustness of immunity. We are also convinced that increasing IGRA may lead to sufficient immunity. Like for other vaccines (e.g. rubellavirus), it would be highly desirable to reach a consensus on laboratory threshold levels as correlates of protection.

### Limitations and restrictions

We are very aware that immunological tests are subject to a number of limitations. Although manifold evidence suggests that there is a correlation between neutralising activity in plasma and protection from symptomatic infection at the population level, the titres of neutralising antibodies decrease over time after infection or vaccination; the kinetics of the decrease vary from person to person. Even normalisation to the WHO standard may not fully compensate for the inter-assay variability of pseudovirus-based neutralisation assays. The neutralization surrogate test used was created with the Wuhan variant and therefore does not correctly reflect the neutralization capacity against emerging SARS-CoV-2 variants. High speed development of variants (of concern) with presumably altered surface properties made the prognosis of protection somewhat difficult, e.g. the shift from Delta to Omicron basically led to a shift from protection against infection to “limited protection against severe sequelae/severe disease”. Consequently this surrogate of protection might (as an absolute value) only hold true for conditions present during the study period. Furthermore, exposure to high viral loads would require higher protective titres than exposure to low viral loads (e.g. when masks are worn).

## Conclusions

Our study offers statements from a time when the Delta variant was predominant. The conclusions do not necessarily apply to other variants such as Omicron. For residents, the mean time between the second vaccination and blood collection was 22 days longer than for HCW (195 days and 173 days, respectively). This could influence the results in such a way that the decline of SARS-CoV-2 S1 reactive T cells and of the neutralisation capacity over time may be overestimated. On the other hand, this bias may be counteracted by our conservative approach to deal with the summary categories for laboratory results above certain thresholds. We substituted such observations with continuous values very close to the respective threshold. It can be assumed that the truncated values are in truth higher and that consequently reductions over time would be more pronounced (sensitivity analyses not shown). Despite the limitations of our study, it is time to shift the consideration from the population level to the individual.

## Abbreviations

- COVID-19: coronavirus disease 19
- ELISA: enzyme-linked immunosorbent assay
- IgG/A: immunoglobulin G/A
- rtPCR: real-time polymerase chain reaction
- SARS-CoV-2: severe acute respiratory syndrome coronavirus 2

## Notes

### Ethics approval and consent to participate

The study was conducted in accordance with the Declaration of Helsinki and Good Clinical Practice [24] and approved by the ethics committee of the University of Luebeck (21-353).

### Availability of data and materials

The datasets used and/or analyzed during the current study are available from the corresponding author on reasonable request.

### Competing interests

The authors declare that they have no competing interests.

### Funding

The study was financed by the City of Luebeck, University Luebeck, Vorwerker Diakonie, Euroimmun AG, and Klinisch-Immunologisches Labor Stoecker.

### First and senior authorship

JS and NE contributed equally to this work and share first authorship. WS and AM contributed equally to this work and share senior authorship.

### Authors’ contributions

JS, NE and HB had full access to all the data in the study and take responsibility for the integrity of the data and the accuracy of data analysis. SJ, KW, SS, CE and DB were responsible for blood collections and appropriate pre-analytic procedures. DB provided the data of the suitable study participants. BT was responsible for laboratory testing. WS and AM developed the study design and supervised the study. All authors contributed to the drafting of the manuscript and agreed with the final version.

### Acknowledgements

The authors would like to thank the patients for sharing their data and all medical and laboratory personnel and administrative staff who contributed to obtain the results. 

This manuscript has been released as a pre-print at medRxiv [25].

## Supplementary Material

Supplementary figures

## Figures and Tables

**Table 1:**
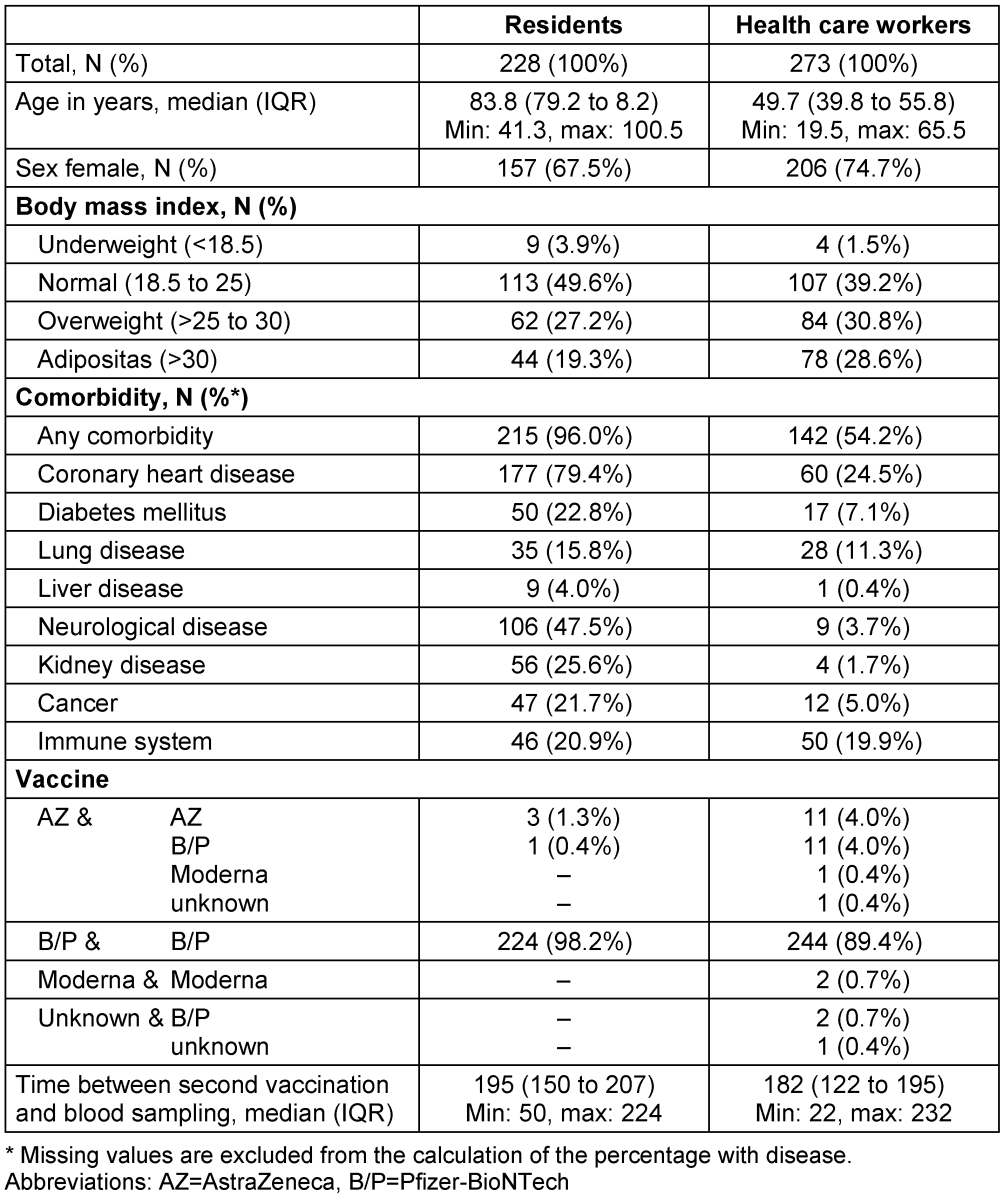
Description of the study population
