# Supplementary material for: Dynamics of immunity over time: decline of anti-SARS-CoV-2 IgG antibodies and T-cell responses after mRNA vaccination in residents and health care workers in nursing homes and homes with assisted living support
Source: GMS Infect Dis. 2023 Sep 6;11:Doc02. doi: 10.3205/id000082 (PMC10565841; doi:10.3205/id000082)
Supplement: Supplementary figures [file ID-11-02-s-001.pdf]

Supplementary figures

Figure S1: Pairwise bivariate scatterplots and Spearman correlation coefficients for anti-SARS-CoV-2 S1-protein IgG Antibodies, neutralisation capacity and interferon-gamma

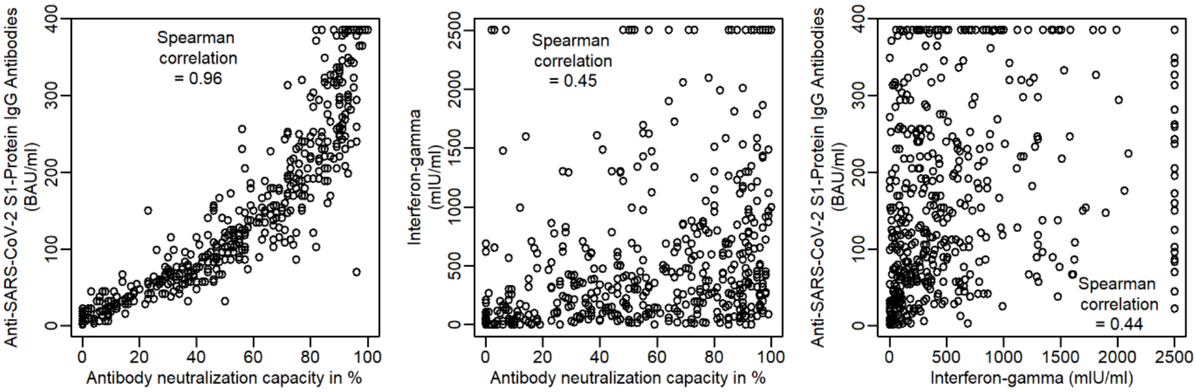

Figure S2: Proportion of persons that are protected against Covid-19 disease over time after second vaccination in the total group by age group and by comorbidity status. A person is considered to be protected against COVID-19 if the SARS-CoV-2 S1 reactive T-cell test is positive, i.e. >200 mIU/ml, and the anti-SARS-CoV-2 S1-protein IgG antibody level is >200 BAU/ml (which corresponds to a neutralisation capacity of >75%). Dots and crosses indicate the individual protection status (0%=not protected, 100%=protected), the line indicates the predicted proportion, and the grey area indicates the 95% prediction band.

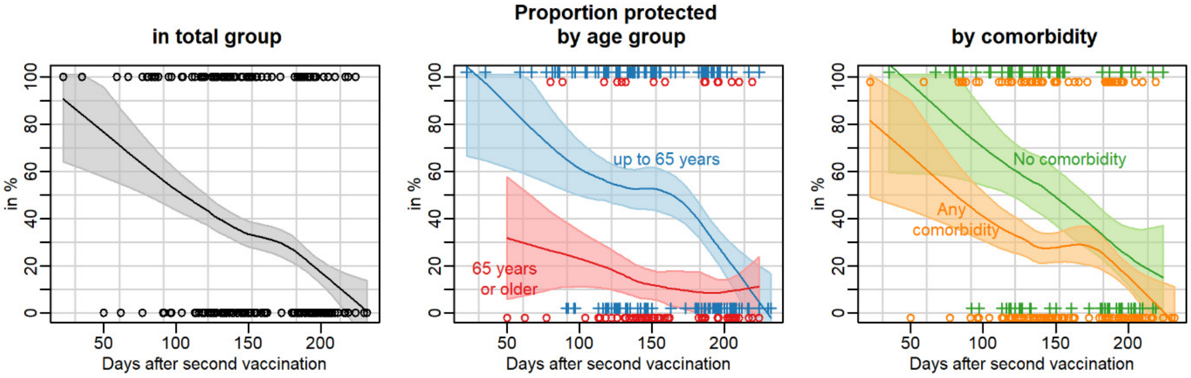

Attachment 1 to Schiffner J, Eisemann N, Baltus H, Jensen S, Wunderlich K, Schuesseler S, Eicker C, Teegen B, Boniakowsky D, Solbach W, Mischnik A. Dynamics of immunity over time: decline of anti-SARS-CoV-2 IgG antibodies and T-cell responses after mRNA vaccination in residents and health care workers in nursing homes and homes with assisted living support. GMS Infect Dis. 2023;11:Doc02. DOI: 10.3205/id000082

Figure S3: Proportion of persons that are protected against Covid-19 disease over time after second vaccination in the total group by age group and by comorbidity status. A person is considered to be protected against COVID-19 if only the anti-SARS-CoV-2 S1-protein IgG antibody level is >200 BAU/ml (which corresponds to a neutralisation capacity of >75%). Dots and crosses indicate the individual protection status (0%=not protected, 100%=protected), the line indicates the predicted proportion, and the grey area indicates the 95% prediction band.

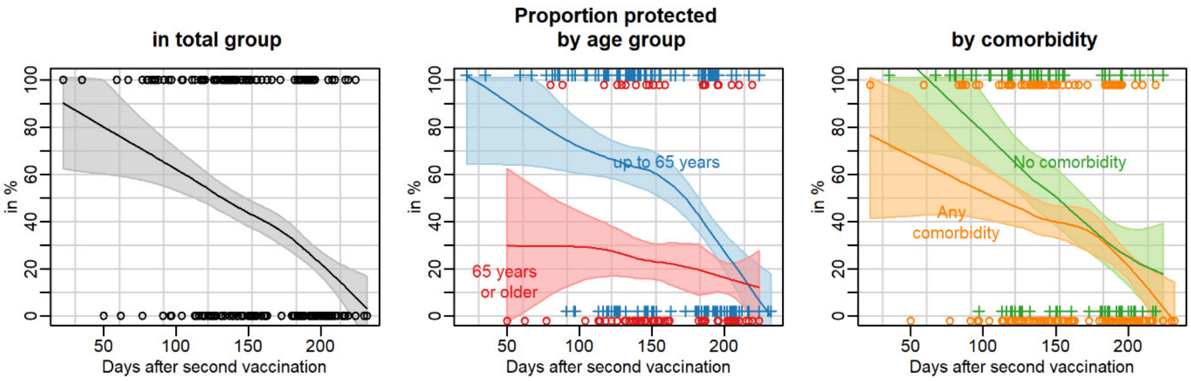

Figure S4: Proportion of persons that are protected against Covid-19 disease over time after second vaccination in the total group by age group and by comorbidity status. A person is considered to be protected against COVID-19 if the anti-SARS-CoV-2 S1-protein IgG antibody level is >264 BAU/ml (threshold from Feng et al. [18]). Dots and crosses indicate the individual protection status (0%=not protected, 100%=protected), the line indicates the predicted proportion, and the grey area indicates the 95% prediction band.

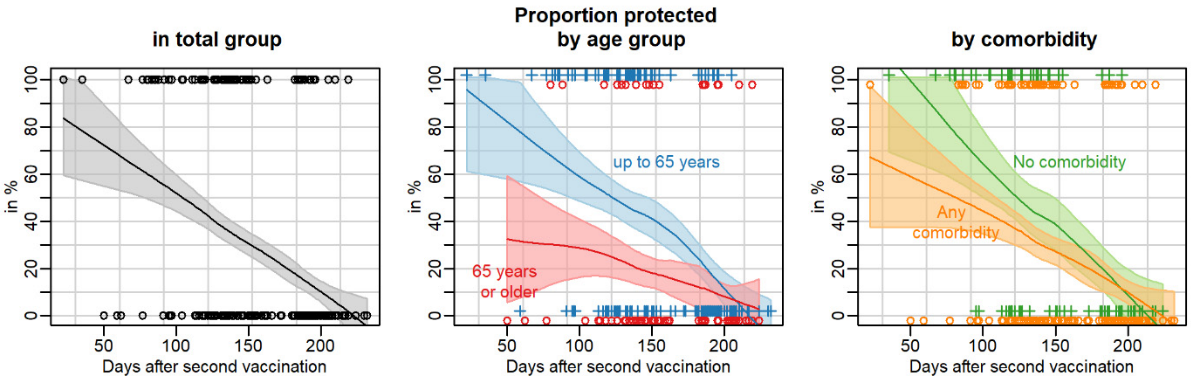

Attachment 1 to Schiffner J, Eisemann N, Baltus H, Jensen S, Wunderlich K, Schuesseler S, Eicker C, Teegen B, Boniakowsky D, Solbach W, Mischnik A. Dynamics of immunity over time: decline of anti-SARS-CoV-2 IgG antibodies and T-cell responses after mRNA vaccination in residents and health care workers in nursing homes and homes with assisted living support. GMS Infect Dis. 2023;11:Doc02. DOI: 10.3205/id000082
